# Acute Abducens Nerve Palsy in a Patient who Sustained Mechanical Trauma to the Orbit

**DOI:** 10.22599/bioj.250

**Published:** 2021-12-09

**Authors:** Adam Geressu, Jayaprakash Patil, Jessica Cody

**Affiliations:** 1 Royal Lancaster Infirmary, GB

**Keywords:** Abducens Nerve, Cranial Nerve Six, Lateral Rectus Palsy, Orbital Fracture, Head Trauma

## Abstract

**Background:**

Abducens nerve (Cranial Nerve VI) innervates the lateral rectus (LR) muscle. Head trauma is one of the most common causes of abducens nerve palsy. Orbital and/or facial injuries could also affect the LR muscle directly or the orbital course of abducens nerve and lead to palsy. We present a case of a young man with multiple orbital fractures and an impingement of the LR muscle resulting in a complete loss of abduction.

**Case Report:**

A 29-year-old male reported falling 15 feet. He presented with diplopia and had complete abduction deficit of the left eye. Orbital CT imaging revealed a bony spur from his left zygomatic bone impinging on the lateral rectus muscle. In view of –4 abduction deficit, he was operated upon to remove the bony spur. This led to a gradual, but complete recovery of his abduction.

**Discussion:**

The abducens nerve has a tortuous course and as a result is commonly injured during head trauma, in particular due to its vulnerability as it passes into Dorello’s canal, or its journey through the cavernous sinus. The case report highlights orbital causes such as direct muscle avulsion or injury to the orbital portion of the abducens nerve, as reasons for how LR weakness could be easily overlooked, unless specifically examined with high-resolution orbital imaging.

**Conclusion:**

Orbital mechanical causes can be overlooked in LR palsy. We emphasise the role of orbital imaging in any patient with abducens nerve or LR Palsy and reaffirm that not all cases are associated with an intracranial cause.

## Introduction

Blunt trauma to the orbital rim is a frequent cause of both orbital fractures and damage to the surrounding facial bones and soft tissues. Many surgical specialties, including orbital surgeons, otolaryngologists, maxillofacial specialists, neurosurgeons, and plastic surgeons, evaluate and treat orbital fractures. Head trauma is one of the most common causes of cranial nerve palsies (Azarmina & Azarmina 2013), with abducens nerve palsy the most common isolated ocular motor palsy (Elder et al. 2016). We describe a case of unilateral abducens nerve palsy due to a fractured fragment impinging on the lateral rectus muscle following a fall.

## Case Report

**Figure 1:**
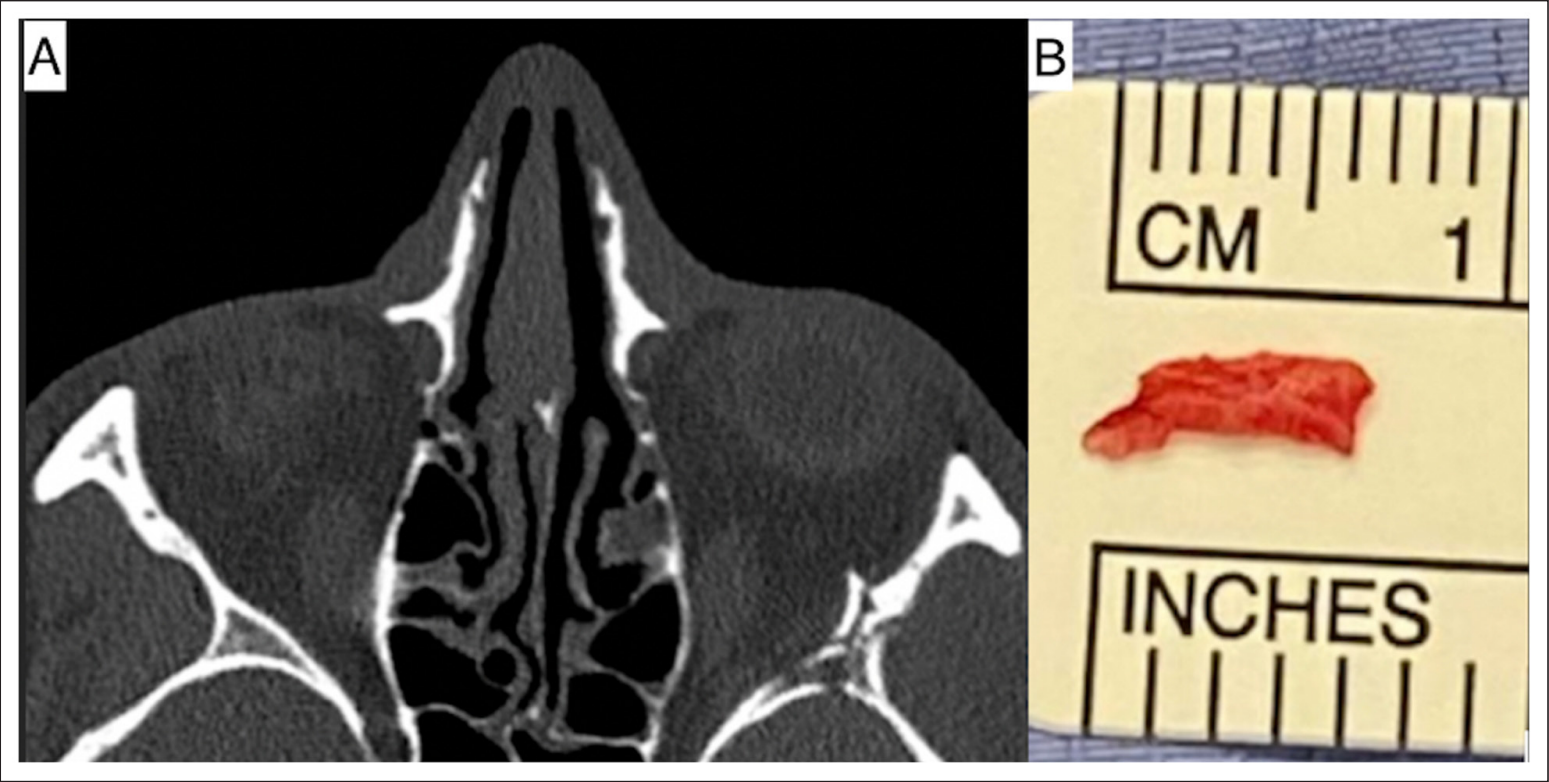
**(A)** Nonenhanced brain CT scan at admission. Bone view showing fracture of the lateral wall of the left orbit (white arrow), with a small area of haemorrhage in the left anterior temporal fossa. **(B)** Bony spur that was removed from lateral rectus muscle.

A 29-year-old was admitted to the emergency department following a 15-foot fall from a roof. As a result of his fall, he sustained multiple fractures as well as a subdural hematoma. On admission he was evaluated with a non-contrast computed tomography (CT) scan. The CT scan revealed fracture of the left orbit at the medial, lateral, and inferior walls. A bony spur from the lateral wall was noted close to the left lateral rectus muscle (***Figure 1A***). There was no detachment or hematoma of the muscle. His other injuries included tripod fractures to the left facial bones, extensive left arm fractures and left iliac crest fracture.

The patient complained of diplopia and was noted to have complete loss of abduction in the left eye. Unaided visual acuity was 6/6 Snellen in both eyes. Intraocular pressures were 15 mm Hg OU. There was left subconjunctival haemorrhage with ecchymoses in the periorbital area. There was no proptosis. The globe was intact, and there was no suggestion of any retrobulbar haemorrhage. A large angle left esotropia was evident in the primary position, with limitations in abduction and depression. Elevation was full. The anterior segment and fundus examination was otherwise unremarkable. His right eye was unaffected.

Further orbital and neuro-MDT meetings suggested the need to remove the bony spur completely as it was now found to be impinging and hooking on the fascial sheath of the lateral rectus, possibly mechanically preventing any abduction. After two weeks, the maxillofacial team removed the bony spur impinging on the lateral rectus muscle (***Figure 1B***) and reconstructed the orbital wall. Intraoperative forced duction test showed no mechanical restriction of eye movements in any gaze. Postoperative follow-up orthoptic assessments, however, did not show any improvement in his lateral rectus functioning.

Because the situation failed to resolve, high-resolution magnetic resonance imaging (MRI) of the orbit, brain and the internal auditory meatus with contrast was performed to further evaluate the left lateral rectus and the course of the left abducens nerve. This revealed mild atrophy of the left lateral rectus muscle. This finding was also corroborated with the inability to find the orbital course of the sixth nerve innervating the lateral rectus.

**Figure 2:**
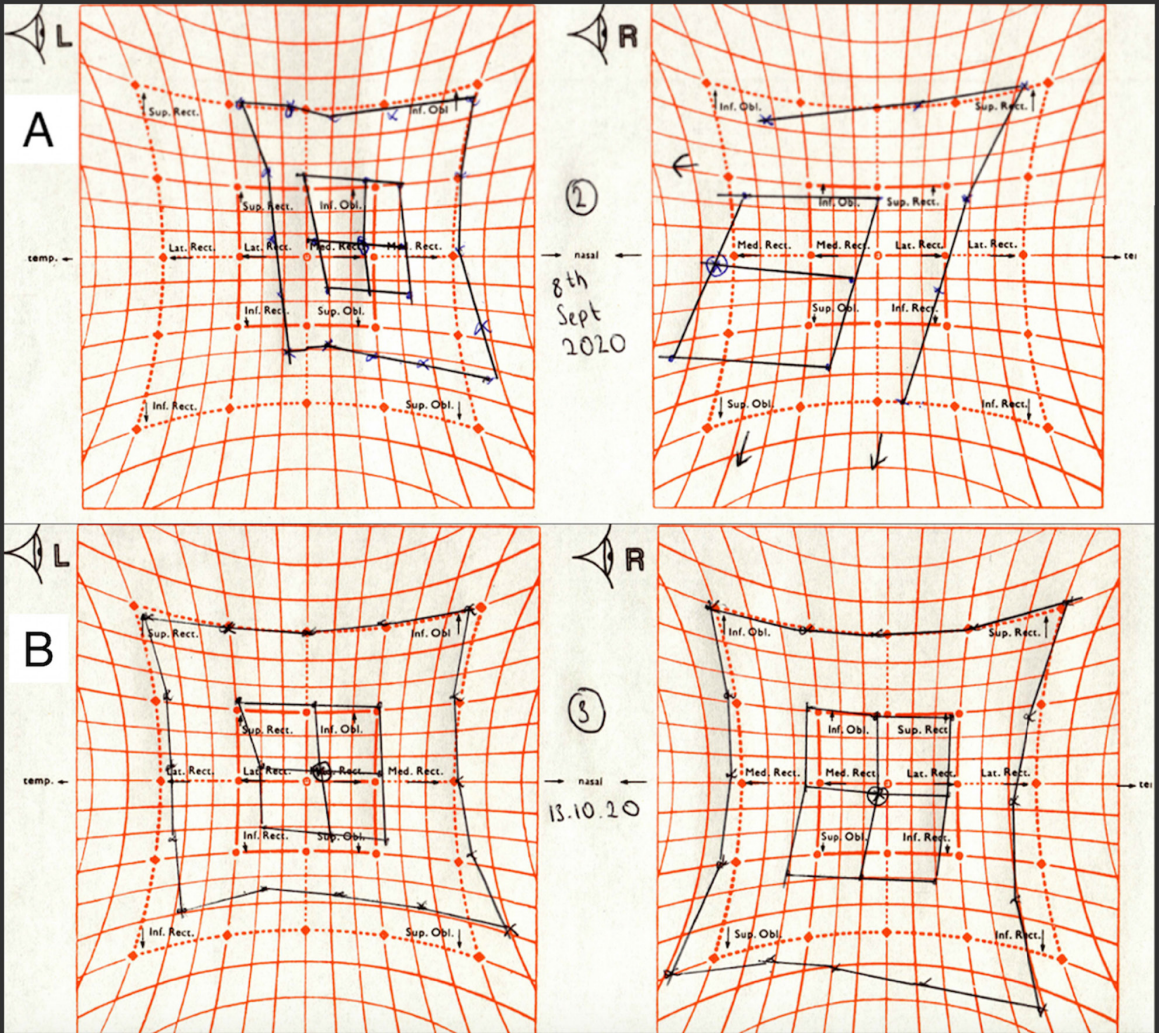
Hess chart demonstrating left abducens nerve palsy with overaction of medial rectus muscle. **(A)** Immediately after surgery and 1 month after presentation. **(B)** 1 month after surgery.

Following this second intervention and three months since onset, the patient showed a slow but marked improvement in abduction (***Figure 2B***). At discharge, there was a small left hyperphoria in the primary position due to residual restriction of the left inferior rectus, and the patient was able to maintain comfortable binocular single vision with a small chin depression.

## Discussion

The abducens nerve is a cranial nerve which has a tortuous course before innervating the ipsilateral lateral rectus muscle (Fam et al. 2015). The abducens nerve emerges from the ponto-medullary junction. It passes through the apex of the petrous temporal bone, making an angle as it enters the cavernous sinus through Dorello’s canal. After that it enters the orbit via the annulus of Zinn through the superior orbital fissure.

Thus, due to these anatomic characteristics of the nerve and its inherent vulnerabilities, paralysis of the abducens nerve is the most common paralysis among all cranial nerves, and commonly injured following head trauma (Keskin et al. 2015; Iaconetta et al. 2007). Dysfunction of the abducens nerve can result from lesions occurring anywhere along its course from the abducens nerve nucleus in the dorsal pons to the lateral rectus muscle within the orbit (Lelli, Milite & Maher 2007).

The incidence of cranial nerve injury following closed head injury varies between 5% and 23% (Pathi et al. 2021), while the reported incidence of unilateral abducens palsy from head trauma is 1–2.7% (Arias 1985). In the current case report, we highlight the role of high-resolution imaging in such orbital and head trauma. Localised orbital causes for abducens nerve palsy cannot be overlooked as in our case report. The young patient here had the bony spur impinging on the muscle sheath that led to the mechanical restriction of the lateral rectus. In addition, the orbital course of the sixth nerve to the muscle was also not visualised, raising the suspicion of the partial or complete severance of the nerve or the nerve fibre bundles. The reduction in the muscle bulk on the affected side indicates the muscle wasting due to denervation.

Approximately half of all abducens nerve palsies recover spontaneously approximately three months after onset. Holmes reported spontaneous recovery following an abducens nerve palsy by two months in 36.6% and by six months in 73.7% (Holmes et al. 2001). In this case, we report recovery commences only after the bony spur was removed, relieving mechanical pressure on the lateral rectus muscle.

Complete palsy is one of predictors for non-recovery (Goodwin 2006), although the underlying cause for this is not clearly understood. Alternative factors should be borne in evaluation while a non-recovery or delayed recovery is encountered. Our case demonstrates unequivocally the mechanical factor from the bony spur and neuropathic factor from the damage to the nerve fibres resulting in the delayed recovery. The mechanical factor was addressed surgically. Any likely damage to the nerve fibrils however resulted in a favourable outcome due to denervation hypersensitivity or nerve growth from the neurotrophic factors.

This also highlights the importance of clearing any loose bony spurs or fragments during the initial orbital or maxillofacial procedures, no matter how miniscule or innocuous they may appear in the preoperative assessment or intraoperatively. As evidenced by our case, the lateral rectus function recovered only following the removal of the bony spur. Contrary to initial radiological impression the bony spur was quite sizeable (8 mm in length) in our case.

One of the limitations of our study is the lack of any neurophysiological investigations in the form of electromyography of the extraocular muscles (EOM-EMG) to confirm the nerve damage and or recovery. This study however is not widely available and, in our case, would have offered limited diagnostic value, although prognostication could have been better.

We also need to realise the limitation of any imaging studies. A multidisciplinary approach would be the way forward in effective management of cases with such a gamut of symptoms.

## Conclusion

Unilateral abducens nerve palsy caused by direct impingement of an orbital bone to the lateral rectus muscle is an unusual presentation. Thorough clinical examination and radiological investigation is required in such cases. CT scan followed by an MRI are excellent investigations to assess cranial nerve injuries in the evaluation of trauma. Inability to abduct eye past the midline and bilateral palsy have a poor prognosis. Once a patient is diagnosed, close follow-up and early intervention may be beneficial to improve prognosis of patient.
